# Supplementary material for: Bamboo-Based Carbon/Co/CoO Heterojunction Structures Based on a Multi-Layer Periodic Matrix Array Can Be Used for Efficient Electromagnetic Attenuation
Source: Materials (Basel). 2024 Oct 28;17(21):5239. doi: 10.3390/ma17215239 (PMC11547459; doi:10.3390/ma17215239)
Supplement: Supplementary file 1 [file materials-17-05239-s001.zip › materials-3238653-supplementary.pdf]

Bamboo-Based Carbon/Co/CoO Heterojunction Structures Based on a Multi-Layer Periodic Matrix Array Can Be Used for Efficient Electromagnetic Attenuation

He Han [1,†], Hui Chen [2,†], Rui Wang [1] and Zhichao Lou [1,2,*]

[1] Jiangsu Co-Innovation Center of Efficient Processing and Utilization of Forest Resources, College of Materials Science and Engineering, Nanjing Forestry University, Nanjing 210037, China; 18805166615@163.com (H.H.); punch.te@icloud.com (R.W.)
[2] Joint International Research Lab of Lignocellulosic Functional Materials, College of Light Industry and Food Engineering, Nanjing Forestry University, Nanjing 210037, China; huichen@njfu.edu.cn
* Correspondence: zc-lou2015@njfu.edu.cn
† These authors contributed equally to this work.

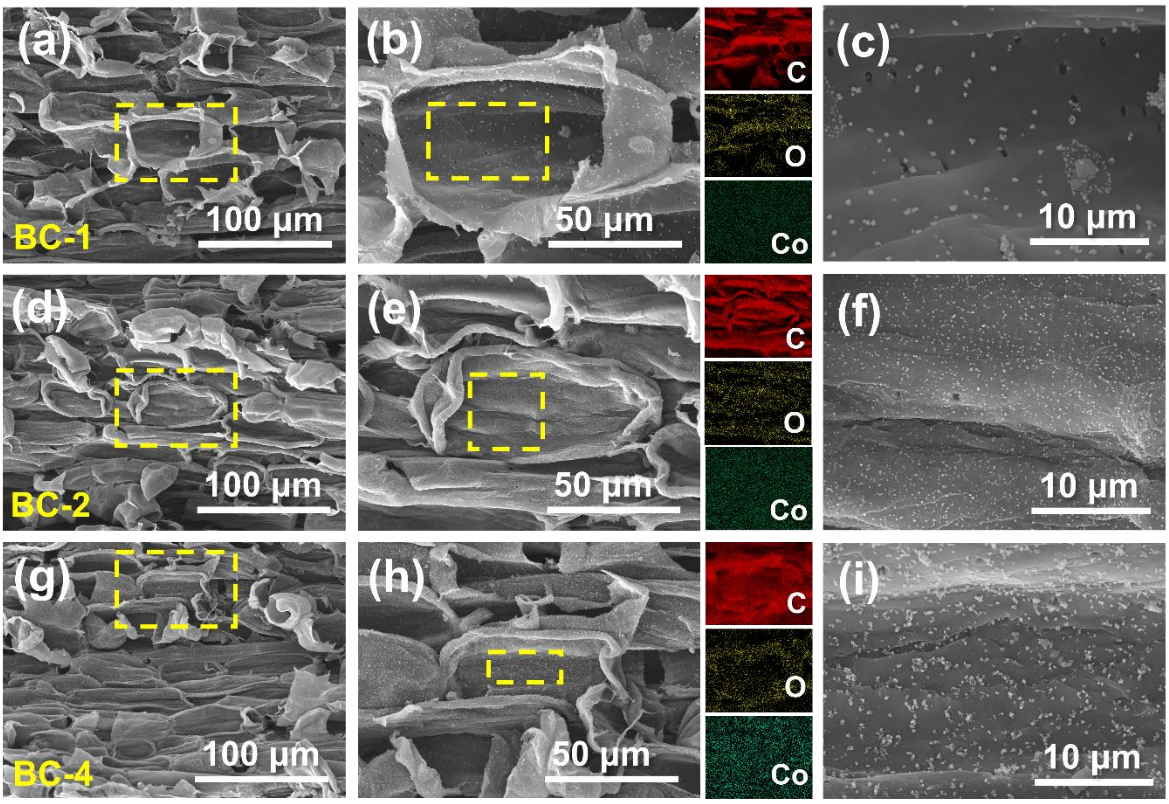

Figure S1. SEM and EDS images of samples BC-1 (**a**–**c**), BC-2 (**d**–**f**), and BC-4 (**g**–**i**).

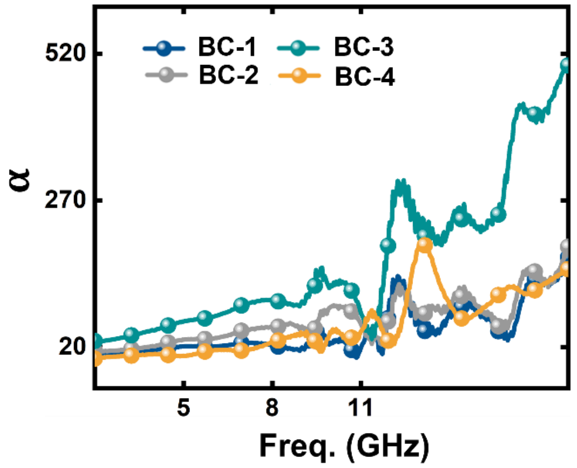

Figure S2. The attenuation constant value of BCs.

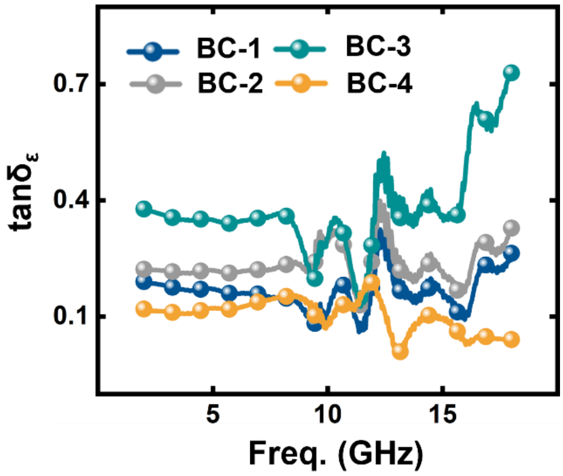

Figure S3. The relationship between the tangent value of the conductivity of the sample and frequency.

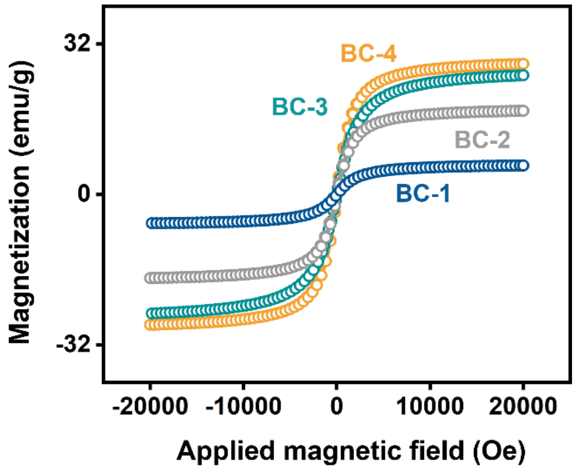

Figure S4. M–H curves of the sample.

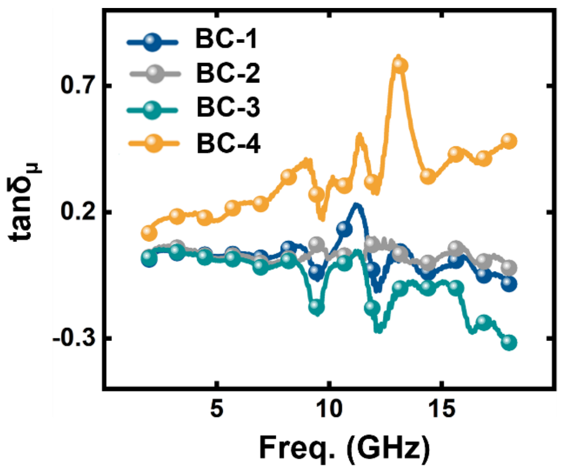

Figure S5. The relationship between the tangent value of the magnetic permeability of the sample and frequency.

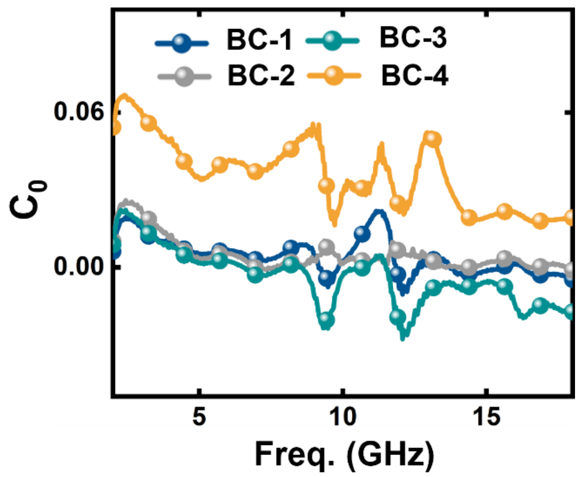

Figure S6. C_0 curves of the sample.

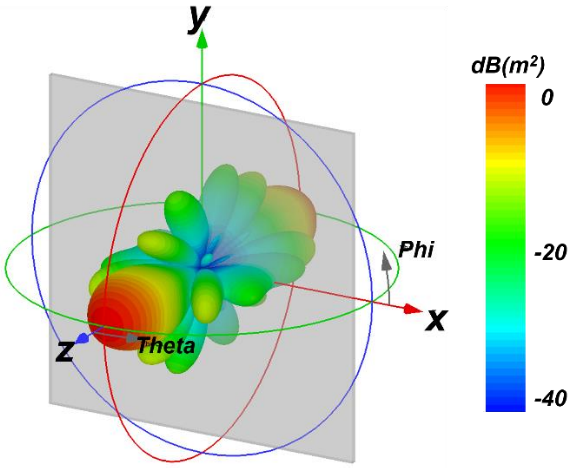

Figure S7. The CST far-field simulation results of PEC.

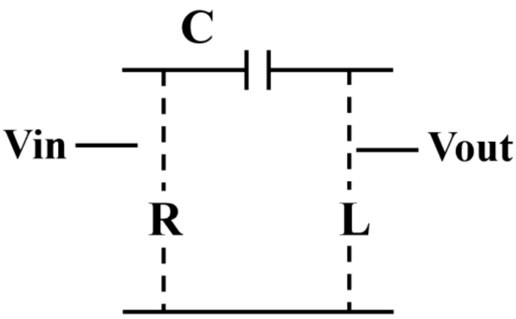

Figure S8. Material Equivalent Circuit Diagram.
